# Integrating DCE‐MRI‐Based Dural Drainage Function Indicators into Machine Learning Models for Improved Intracranial Tumor Prognosis

**DOI:** 10.1002/mco2.70992

**Published:** 2026-09-25

**Authors:** Lusen Ran, Wenxi Luo, Luyun You, Wenjie Wei, Yuqin He, Shabei Xu, Jiayu Zhu, Fan Long, Xiaopeng Song, Guangyuan Hu, Xianglin Yuan, Wei Wang, Feng Lu, Minghuan Wang, YingYing Wu

**Affiliations:** ^1^ Department of Neurology Tongji Hospital Tongji Medical College Huazhong University of Science and Technology Wuhan China; ^2^ College of Life Science and Technology Huazhong University of Science and Technology Wuhan China; ^3^ Department of Neurology The Second Affiliated Hospital of Nanchang University, Nanchang Jiangxi China; ^4^ Department of Oncology Tongji Hospital Tongji Medical College Huazhong University of Science and Technology Wuhan China; ^5^ Central Research Institute United Imaging Healthcare Group Shanghai China; ^6^ Paul C. Lauterbur Research Centre for Biomedical Imaging Shenzhen Institute of Advanced Technology Chinese Academy of Science Shenzhen Guangdong China; ^7^ Shenzhen College of Advanced Technology University of Chinese Academy of Sciences Shenzhen Guangdong China; ^8^ Wuhan Zhongke Industrial Research Institute of Medical Science Wuhan China; ^9^ National Engineering Research Center for Big Data Technology and System Services Computing Technology and System Lab Cluster and Grid Computing Lab School of Computer Science and Technology Huazhong University of Science and Technology Wuhan China

**Keywords:** cohort study, intracranial malignant tumors, machine learning, meningeal lymphatic vessels, survival

## Abstract

Meningeal lymphatic vessels (mLVs) are crucial in intracranial tumor progression. This study investigated whether incorporating mLVs functional indicators into machine learning models enhances prognostic prediction for intracranial malignant tumors. We prospectively enrolled 246 patients, assessing baseline mLVs function via dynamic contrast‐enhanced MRI. After 2.5 years’ follow‐up, 100 patients (51 survivors, 49 deceased) were finally included. The mean area under the receiver operating characteristic curve (AUROC) of the XGBoost model excluding DCE‐MRI‐based dural drainage function indicators (DDFIs) was 0.746 (95% CI: 0.621–0.871), which increased to 0.808 (95% CI: 0.733–0.883) with the inclusion of DDFIs. Kaplan–Meier survival curves demonstrated significantly better discrimination when DDFIs were included (*p* = 5.66 × 10^−8^ vs. p = 1.22 × 10^−4^). The c‐index of the Cox regression model excluding DDFIs was 0.919 (95% CI: 0.916–0.940), rising to 0.948 (95% CI: 0.946–0.955) with their inclusion. In the glioma subgroup (*n* = 43), AUROC rose from 0.804 (95% CI: 0.629–0.980) to 0.904 (95% CI: 0.717–1.000). These findings indicate that integrating mLVs function significantly refines long‐term prognostic accuracy in intracranial malignant tumors, supporting its potential clinical utility.

## Graphical abstract

We integrated DCE‐MRI‐based dural drainage function indicators (DDFIs) that reflect meningeal lymphatic vessels (mLVs) function into XGBoost models to predict the prognosis of intracranial malignant tumors. In a 2.5‐year follow‐up cohort of 100 patients, DDFIs remarkably improved the model's predictive performance, with a more significant enhancement observed in the glioma subgroup. DDFIs are promising novel imaging biomarkers for the prognostic evaluation of intracranial malignant tumors.

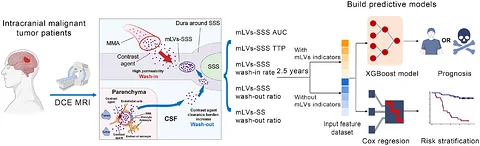

## 1 Introduction

Intracranial malignant tumors represent a significant global health burden, with an estimated 330,000 new cases reported in 2016 [1] and high rates of morbidity and mortality. Among these, glioblastoma multiforme (GBM) is the most common and aggressive subtype, despite standard care, the median survival remains limited to 12–18 months [2, 3]. However, the prognosis of gliomas varies considerably [4], even among patients with the same tumor grade, with some instances of long‐term survival [5]. Developing an accurate progression and survival model for intracranial malignant tumor patients could facilitate personalized treatment planning and optimizing patient management [6, 7, 8]. In recent years, advances in machine learning have yielded superior performance compared to traditional prognosis prediction models [9]. While studies have demonstrated the high predictive value of multimodal imaging, genomics, and metabolomics for glioma prognosis through machine learning algorithms [10, 11, 12], the high cost and complexity of acquiring such data often limit their clinical utility. Therefore, enhancing model accuracy using accessible clinical measures and identifying novel, cost‐effective prognostic markers remain critical research priorities [7].

The discovery of meningeal lymphatic vessels (mLVs) called for a revision of the traditional conception that the central nervous system (CNS) entirely lacks a lymphatic system [13, 14]. The mLVs serve as a pathway for draining macromolecules and immunologically active cells from the cerebrospinal fluid (CSF), meninges, and parenchyma to the deep cervical lymph nodes (dcLNs) [15, 16]. In preclinical glioma models, mLVs transport tumor antigens from the CNS to cervical lymph nodes (CLNs), where they subsequently stimulate specific populations of T‐cells [17]. Furthermore, the function of mLVs has been shown to support the efficacy of programmed cell death protein 1 (PD‐1) therapy and radiotherapy for intracranial tumors in animal models [17, 18, 19, 20]. In our recent clinical investigation with dynamic contrast‐enhanced magnetic resonance imaging (DCE‐MRI), we identified disturbances of mLVs function in intracranial malignant tumor patients, which were directly correlated with the rate of tumor progression [21]. Thus, we conclude that mLVs function may play a significant role in the progression and long‐term prognosis of patients with intracranial malignant tumor.

Given this background, we hypothesize that incorporating mLVs function into a prediction model could improve its prognostic accuracy. To test this hypothesis, we followed a cohort of patients with intracranial malignant tumors for approximately three years. We then constructed a machine learning model to retrospectively predict the prognosis of these patients, using a combination of basic brain imaging features and blood biomarkers. Finally, we evaluated the impact of incorporating mLVs function indicators derived from DCE‐MRI (specifically, DCE‐MRI‐based dural drainage function indicators, or DDFIs) into the prognostic model.

We then developed a machine learning model to retrospectively predict outcomes using a combination of neuroimaging and blood‐based biomarkers. Finally, we evaluated the impact of incorporating DDFIs to determine their added value in prognostic prediction.

## 2 Methods

### 2.1 Patient Selection

**FIGURE 1:**
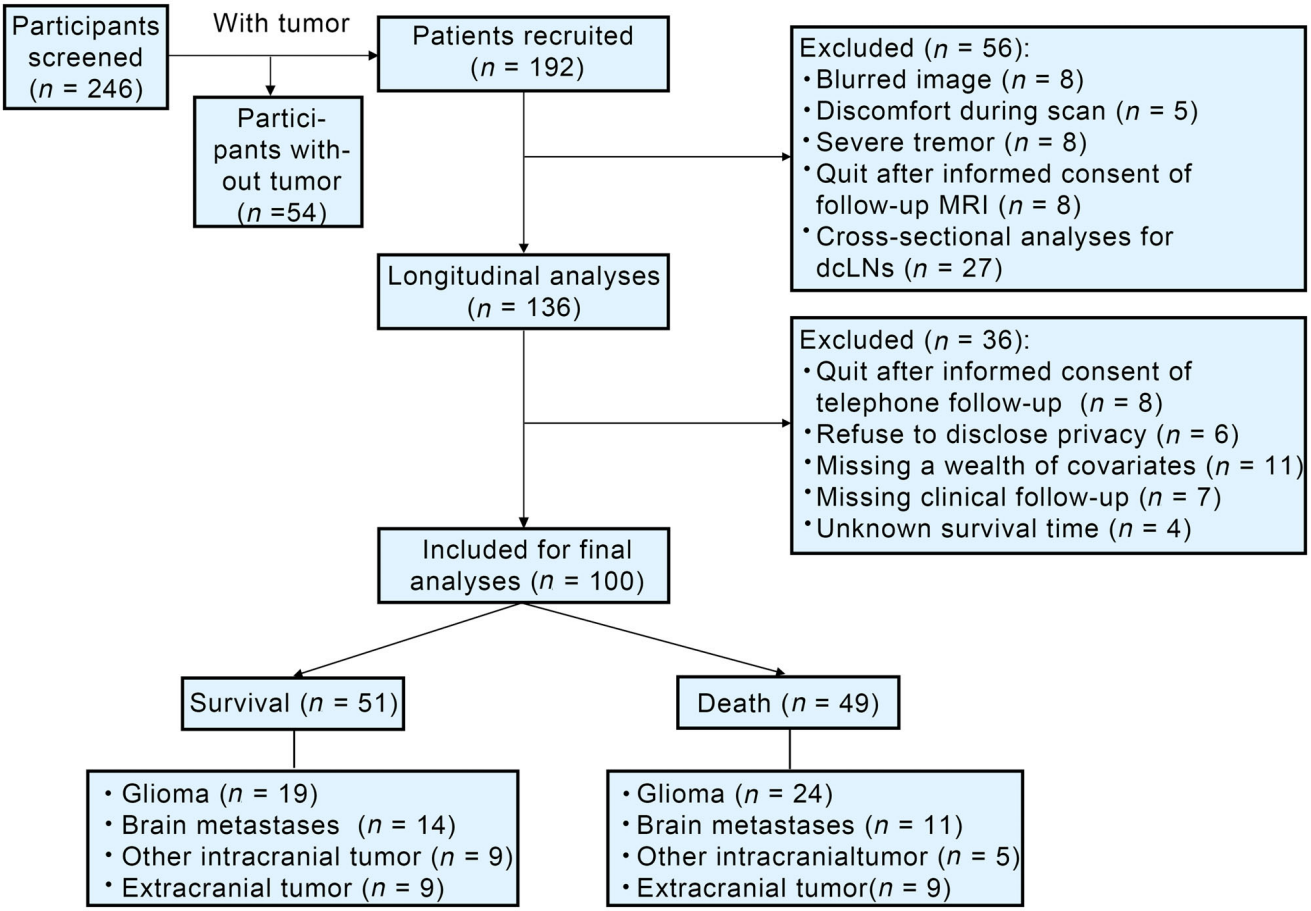
Participant screening flowchart of the study.

All participants provided written informed consent forms before enrolment (Figure 1). Inclusion criteria were as follows: (I) over 18 years of age; (II) available for MRI; (III) creatinine elution more than 60 mL/min; (IV) no history of contrast agent allergy; (V) no diagnosis of other organic diseases of the nervous system unrelated to the tumor; (VI) voluntary consent to participate; and (VII) availability of complete clinical, demographic and follow‐up information. The exclusion criteria were as follows: (I) claustrophobia or other psychiatric disorders; (II) metal implants such as nail plates, insulin pumps, vascular stents, or dentures; (III) diagnosis of any disease of the nervous system; (IV) pregnant or lactating women; and (V) incomplete clinical data or loss to follow‐up.

### 2.2 Clinical Information

Clinical information recorded included basic information, blood tests, and tumor‐related variables. Basic information included patient demographics (age, sex, height, weight, etc.), prior history and treatment (i.e., prior surgery, chemotherapy, radiation therapy). Blood work included routine tests of liver function, renal function, electrolytes, and coagulation indicators. Tumor‐related variables included tumor category, World Health Organization (WHO) grade of glioma, and postoperative recurrence. Tumor category and WHO grade of glioma were determined based on biopsy and imaging. The secondary outcome (intracranial tumor progression) was defined as the appearance of new lesions or an increase of at least 25% in the sum of the longest diameters of existing lesions on contrast‐enhanced T1‐weighted MRI, according to Response Assessment in Neuro‐Oncology (RANO) 2.0 [22].

### 2.3 Imaging Procedure

All MRI scans were acquired on a 3T MRI (uMR 790, United Imaging Healthcare, Shanghai, China), using a 32‐channel radio frequency head array coil for signal reception. Contrast agent Gadobutrol (Gadovist, Bayer Pharma AG) was injected intravenously via an intravenous injection pump at a dose of 0.1 mmol/kg and a rate of 3 mL/s.

**FIGURE 2:**
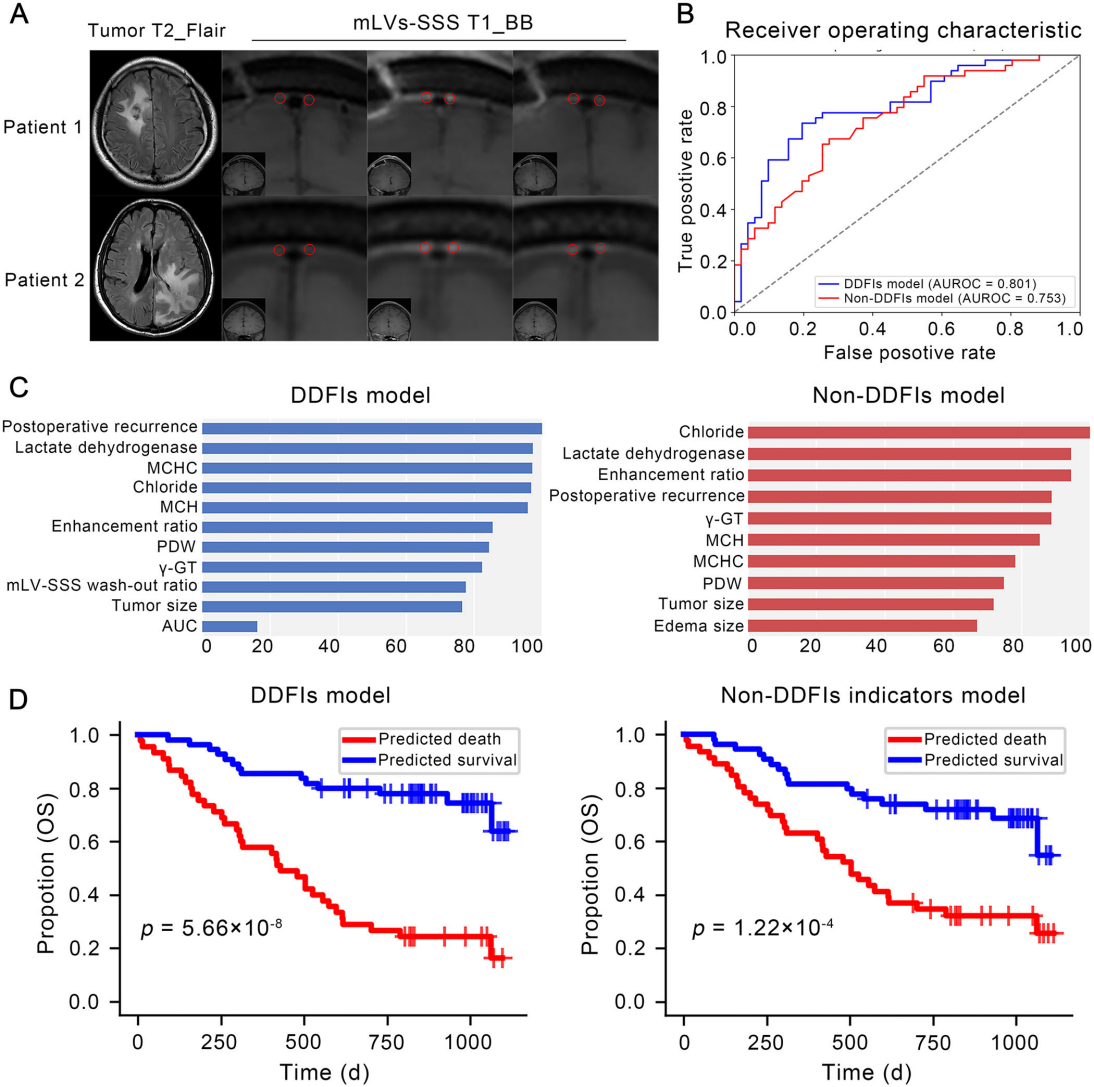
XGBoost model results of all participants. (A) A comparison of mLVs‐SSS wash‐out function in two grade IV glioma patients. At the 3 h point, patient 2 (deceased) had more contrast retention of the agent in mLVs‐SSS than did patient 1 (alive). (B) ROC curves of the XGBoost model with (blue) and without (red) DDFIs. (C) Top ten importance of the characteristics in the XGBoost model with (left) and without (right) DDFIs. (D) KM curves of the XGBoost model with (left) and without (right) DDFIs. Red curve predicts death and blue curve predicts survival. AUC, area under the curve; AUROC, area under the receiver operator characteristic curve; BB, black blood; KM curve, Kaplan–Meier curve; MCH, mean corpuscular hemoglobin; MCHC, mean corpuscular hemoglobin concentration; mLVs, meningeal lymphatic vessels; mLVs‐SSS, superior sagittal sinus mLVs; PDW, platelet distribution width; γ‐GT, gamma‐glutamyl transferase activity; XGBoost, eXtreme gradient boosting.

As detailed in our previous studies, we used T1_black blood (BB) sequences for evaluation of mLVs function [21, 23]. In brief, the mLVs around superior sagittal sinus (mLVs‐SSS) and mLVs around sigmoid sinus (mLVs‐SS) are clearly visible in the gadobutrol enhanced T1_BB sequence. DCE‐MRI examination was used to acquire the time‐signal intensity curve of contrast agent washing into mLVs‐SSS. We performed 20 repetitions of a coronary 2D T1_BB sequence to measure the dynamics of mLVs‐SSS signal intensity during gadobutrol influx, with time resolution of about 17 s per phase. Each repetition contained five continuous slices, and the first image slice was positioned to cut across the anterior margin of the corpus callosum and perpendicular to the SSS. To evaluate the wash‐out kinetics of mLVs‐SSS and mLVs‐SS, we performed three coronary 3D T1_BB sequences (baseline, and 10 min and 3 h after enhancement) (Figure 2A).

The parameters of 2D T1_BB were as follows: TR = 386 ms; TE = 11.2 ms; excitation angle = 90°; refocus angle = 120°; slice number = 5; slice thickness = 3.0 mm; field of view = 170 × 170 mm^2^; matrix = 256 × 256; voxel size = 0.66 × 0.66 × 3 mm^3^; number of repetitions = 20. The parameters of 3D T1_BB were as follows: TR = 750 ms; TE = 19.4 ms; slice number = 150; slice thickness = 1.3 mm; field of view = 216 × 180 mm^2^; matrix = 288 × 240; voxel size = 0.75 × 0.75 × 1.3 mm^3^.

### 2.4 Imaging Analysis

For evaluation of the mLVs‐SSS wash‐in function, we designated three parameters: area under the curve (AUC, representing the integral of the time‐signal intensity curve), time to peak (TTP, representing the time from start of the contrast agent infusion to the point of maximum signal intensity) and wash‐in rate (depicting the maximum slope of the increasing time‐signal intensity curve). After motion correction of the continuous 2D T1_BB sequences, the signal intensities (the highest intensity slice) of left and right mLVs‐SSS were identified by concord of two experienced radiologists to obtain the time‐signal intensity. The above parameters were calculated on the UIH Workstation (uWS‐MR: R005).

For mLVs‐SSS and mLVs‐SS wash‐out function evaluation, we defined the wash‐out ratio as the relative decline in signal intensity at 3 h compared to 10 min, using the formula:

(S_10min_ – S_3h_) / (S_10min_ – S_baseline_)

 where the S_baseline_, S_10 min_, and S_3 h_ represent the signal intensity of mLVs before enhancement and at 10 min, and 3 h after enhancement, respectively. The 3D T1_BB sequence was processed in an automatic analysis pipeline tool based on FMRIB Software Library (FSL). Two experienced radiologists used ITK‐SNAP software to select the coronary position near the anterior margin of the corpus callosum to record the mLVs‐SSS signal intensity, and the coronary position near the posterior margin of the corpus callosum to record the mLVs‐SS, across five successive slices. Signal intensity was corrected by the white matter signal intensity at the same level.

Using the ITK‐SNAP, tumor‐related radiologic measures were assessed by clinical examination. The tumor size, edema size and apparent diffusion coefficient (ADC) signal were evaluated in T1, T2, diffusion‐weighted imaging (DWI) and ADC sequences by the two experienced radiologists. The enhancement ratio was defined as the ratio of average signal of tumor to that of the center of the semiovale white matter in the enhanced T1 sequence. The perivascular space (PVS) rating according to Potter's method on the T2 sequence [24, 25] and it included the following four regions: centrum semiovale, basal ganglia, hippocampus and midbrain.

### 2.5 XGBoost Model

To assess the impact of DDFIs on the prognostic analysis in patients with intracranial malignant tumors, we used the XGBoost (eXtreme Gradient Boosting) algorithm to construct predictive models based on various combinations of these indicators, followed by a comparison of the models' predictive performance. To ensure consistency across variables, we applied scaling to the continuous variables, thus standardizing them to a uniform scale. Initially, we developed a machine learning model using the XGBoost algorithm, incorporating all potential candidate variables to predict clinical outcomes. The importance of each variable was evaluated using information gain, and the variables were ranked accordingly based on their contributions to the model's predictive performance. Based on these importance rankings, variables were sequentially incorporated into the model, creating a series of progressive classifiers.

To ensure both the simplicity and accuracy of the model, we employed an importance‐driven feature selection strategy within a ten‐fold cross‐validation framework to minimize the risk of overfitting and thereby confirm the final combination of indicators. For the XGBoost models, feature importance was quantified based on information gain. Specifically, we identified the cross‐validation fold that achieved the highest AUROC and retained only those indicators with a gain score greater than zero for the final model. Based on the selected indicators, we developed various machine learning models, including those with and without DDFIs. In addition, we used a random search to determine the optimal parameters for each model in the training set. Similarly, for the development of the two machine learning models, we applied a strict ten‐fold cross‐validation framework. All preprocessing steps, including feature scaling, feature selection, and hyperparameter optimization via random search, were performed exclusively within the training folds of each cross‐validation split to prevent data leakage. To evaluate the reliability of different machine learning models, we computed the area under the receiver operating characteristic curve (AUROC) and the area under the precision‐recall curve (AUPRC) for each cross‐validation trial. The final evaluation metric was the average of the results from the ten experiments, which allowed us to compare the performance of different models. Model performance was summarized using AUROC and PR‐AUC. To quantify the added predictive value of DDFIs, paired models were constructed using identical pipelines, differing only in the inclusion of DDFIs. The comparative trend between these models, rather than the absolute magnitude of performance metrics, was used to evaluate the incremental contribution of DDFIs to prognostic modeling.

To further assess the potential clinical utility of the XGBoost‐based prediction models, we performed decision curve analysis (DCA) based on the predicted risk scores generated by the final XGBoost models (Figures S1–S3). DCA evaluates the net benefit across a range of clinically relevant threshold probabilities and is particularly suitable for comparing the relative decision value of competing prediction models without requiring fully calibrated absolute risk estimates. The net benefit of the XGBoost model incorporating DDFIs was compared with that of the model excluding DDFIs, as well as default strategies (treat‐all and treat‐none). As sensitivity analyses, we repeated the entire evaluation using twenty‐fold cross validation and leave one out cross validation, while maintaining the same within split preprocessing, feature selection and hyperparameter tuning procedures. The corresponding AUROC and AUPRC, together with paired differences between models with and without DDFIs, are summarized in Table S1.

### 2.6 Cox Regression Model

For the prognostic analysis of participants, we employed a linear Cox proportional hazards model with L2 regularization (*c* = 0.5). As a robustness check, we repeated the multivariable Cox analysis using a stronger penalization setting and obtained essentially unchanged coefficients and conclusions. To ensure model parsimony and focus on clinical relevance, we implemented a univariate filter for feature selection. Specifically, each candidate indicator was fitted into an individual univariate Cox proportional hazards model using the Python lifelines package. Only features demonstrating statistical significance at the 95% confidence level (*p* < 0.05) were selected as input variables for the final multivariate model. Univariate and multivariate Cox proportional hazards regression analyses were conducted to identify potential prognostic factors. Kaplan–Meier (KM) analysis was used to determine whether each model could stratify patients into clinically meaningful groups. Group membership was defined based on the median of the model's predicted outcomes, serving as the threshold for categorization. To compute *p*‐values for the concordance index (*c*‐index), we used 1000‐fold permutation testing. The 95% confidence intervals (CIs) for the *c*‐index were estimated using 100‐fold leave‐one‐out bootstrap resampling. All *p*‐values for the KM analyses were calculated using the multivariable log‐rank test, and we reported the significance of covariates in the Cox proportional hazards model with *c* = 0.5. Lastly, survival rates were estimated using linear interpolation.

### 2.7 Statistical Analyses

Statistical analyses were performed via IBM SPSS Statistics 25.0 and Python v.3.8.3. All graphs were generated using Python v.3.8.3. Analyses in Python v.3.8.3 were conducted using custom code written with the following libraries: Pandas v.1.5.0, NumPy v.1.23.4, Lifelines v.0.27.4, Matplotlib v.3.5.2, Seaborn v.0.11.2, SciPy v.1.9.1, scikit‐learn v.1.3.2, and Joblib v.1.2.0. For continuous data, a normality test was performed. If all groups satisfied normality and showed equal variance between two groups, we used a two‐sample *t*‐test for between‐group comparison; otherwise, we used a Mann–Whitney *U*‐test. For categorical data, we used the chi‐square test. Normally distributed continuous variables are expressed as mean (Standard Deviation, SD), non‐normally distributed ones as median [Interquartile Range, IQR]. Categorical variables are shown as frequency (percentage). For KM survival analysis, the log‐rank test was applied to assess the statistical significance between groups. A *p*‐value less than 0.05 was considered statistically significant. Further details of XGBoost and Cox regression are provided above.

## 3 Results

### 3.1 Demographic and Clinical Characteristics of Patients

A total of 246 participants were screened from the Physical Examination Center and the Oncology Department of Tongji Hospital between March 2021 and September 2022. Of these, 192 patients with tumors were enrolled in the longitudinal study, with 136 remaining after initial exclusions (Figure 1). After a 2.5‐year follow‐up, 36 additional patients were excluded and 100 patients were left in the final analysis, of whom 51 were alive and 49 had died by the end of the follow‐up period. The cohort had a mean (SD) age of 47.0 (13.3) years, with 61% being male. The mean (SD) follow‐up duration was 30.4 (4.8) months. For DDFIs, mean (SD) values were: AUC of 2636.6 (678.7) A.U.×s, TTP of 143.1 (39.2) s, wash‐in rate of 3.56 (1.16) A.U./s, mLVs‐SSS wash‐out ratio of 0.68 (0.12), and mLVs‐SS wash‐out ratio of 0.67 (0.11). Significant differences between survivors and deceased patients were observed for several variables, including sex (*p* = 0.012), postoperative recurrence (*p* = 0.002), WHO glioma grade (*p* = 0.017), edema size (*p* = 0.036), LDH (*p* = 0.009), MCH (*p* = 0.003), MCHC (*p* = 0.004), platelet distribution width (PDW) (*p* = 0.027), γ‐GT (*p* = 0.011), chloride (*p* = 0.004), and mLVs‐SSS wash‐out ratio (*p* = 0.016) (Table 1). These findings highlight the potential prognostic value of DDFIs and other clinical variables in predicting patient outcomes.

**TABLE 1:** Baseline characteristic of the participants.

	Total (*n* = 100)^a^	Survived (*n* = 51)	Deceased (*n *= 49)	*p* ^b^
**Demographic information**				
Sex, *n* (%)				0.012
Male	61 (61.0)	25 (49.0)	36 (73.5)	
Female	39 (39.0)	26 (51.0)	13 (26.5)	
Age, year	47.0 (13.3)	46.6 (13.9)	47.5 (12.8)	0.720
Height, m	1.65 [0.12]	1.63 [0.12]	1.68 [0.07]	0.083
Weight, kg	63.5 (11.2)	63.0 (10.9)	63.9 (11.6)	0.669
Follow‐up time, days	923.5 [217.0]	923.0 [223.0]	924.0 [227.0]	0.898
**Tumor indicators**				
WHO grade of glioma, *n* (%)				0.017
II	16 (37.2)	11 (57.9)	5 (20.8)	
III	5 (11.6)	3 (15.8)	2 (8.3)	
IV	22 (51.2)	5 (26.3)	17 (70. 8)	
Other tumor (glioma excluded)	57 (100.0)	32 (56.1)	25 (43.9)	0.236
Postoperative recurrence, *n* (%)				0.002
Yes	27 (27.0)	7 (13.7)	20 (40.8)	
No	32 (32.0)	23 (45.1)	9 (18.4)	
Denial of craniotomy	41 (41.0)	21 (41.2)	20 (40.8)	
Tumor size, mm^3^	739.0 [1168.5]	946.0 [1063.7]	562.0 [1179.9]	0.505
Edema size, mm^3^	1488.3 [2547.1]	1127.6 [2011.0]	2668.2 [2566.8]	0.036
ADC signal	1390.7 [312.4]	1354.1 [345.4]	1394.5 [274.5]	0.347
Enhancement ratio	1.44 (0.44)	1.49 (0.57)	1.41 (0.33)	0.586
**Blood tests**				
Lymphocyte percentage, %	27.3 (9.4)	29.2 (10.1)	25.2 (8.2)	0.032
LDH, U/L	169.0 [59.0]	160.0 [43.0]	185.0 [63.3]	0.009
D‐Dimer, µg/mL FEU	0.37 [0.57]	0.38 [0.46]	0.34 [1.14]	0.823
MCV, fL	91.6 [5.9]	90.8 [6.6]	92.2 [6.3]	0.067
MCH, pg	30.8 [2.6]	30.3 [2.7]	31.1 [2.5]	0.003
MCHC, g/L	335.3 (10.3)	332.5 (9.0)	338.3 (10.8)	0.004
RDW‐CV	13.2 [2.0]	13.1 [1.7]	13.2 [2.3]	0.801
PDW, fL	11.9 [3.5]	12.7 [3.4]	11.4 [2.9]	0.027
**Blood tests**				
MPV, fL	10.4 [1.6]	10.6 [1.7]	10.1 [1.3]	0.025
P‐LCR, %	29.5 (8.9)	31.6 (9.4)	27.5 (8.0)	0.021
Total bilirubin, µmol/L	7.90 [5.80]	7.30 [4.40]	8.70 [8.13]	0.119
Direct bilirubin, µmol/L	3.10 [1.80]	3.00 [1.40]	3.65 [2.28]	0.059
γ‐GT, U/L	23.0 [18.0]	19.0 [17.0]	25.0 [29.0]	0.011
Chloride, mmol/L	103.4 (2.5)	104.1 (2.2)	102.7 (2.5)	0.004
**DDFIs**				
AUC, A.U. × s	2636.6 (678.7)	2579.5 (706.3)	2696.1 (650.6)	0.393
TTP, s	143.1 (39.2)	139.5 (40.0)	146.9 (38.4)	0.346
Wash‐in rate, A.U. / s	3.56 (1.16)	3.48 (1.15)	3.63 (1.18)	0.527
mLVs‐SSS wash‐out ratio	0.68 (0.12)	0.71 (0.10)	0.65 (0.13)	0.016
mLVs‐SS wash‐out ratio	0.67 (0.11)	0.69 (0.11)	0.65 (0.10)	0.106

Abbreviations: ADC, apparent diffusion coefficient; AUC, area under the curve; DDFIs, DCE‐MRI‐based dural drainage function indicators; LDH, lactate dehydrogenase; PDW, platelet distribution width; P‐LCR, platelet large cell ratio; RDW‐CV, red cell distribution width ‐ coefficient of variation; TTP, time to peak; MCH, mean corpuscular hemoglobin; MCHC, mean corpuscular hemoglobin concentration; MCV, mean corpuscular volume; mLVs‐SS, sigmoid sinus mLVs; mLVs‐SSS, superior sagittal sinus mLVs; MPV, mean platelet volume; γ‐GT, gamma‐glutamyl transferase; WHO, World Health Organization.

^a^ Data were presented as mean ± (standard deviation), median [Interquartile Range] or *n* (percentage).

^b^ Obtained by independent sample *t*‐test, chi‐square test or non‐parameter test.

### 3.2 DDFIs Improve XGBoost's Predictive Performance

**FIGURE 3:**
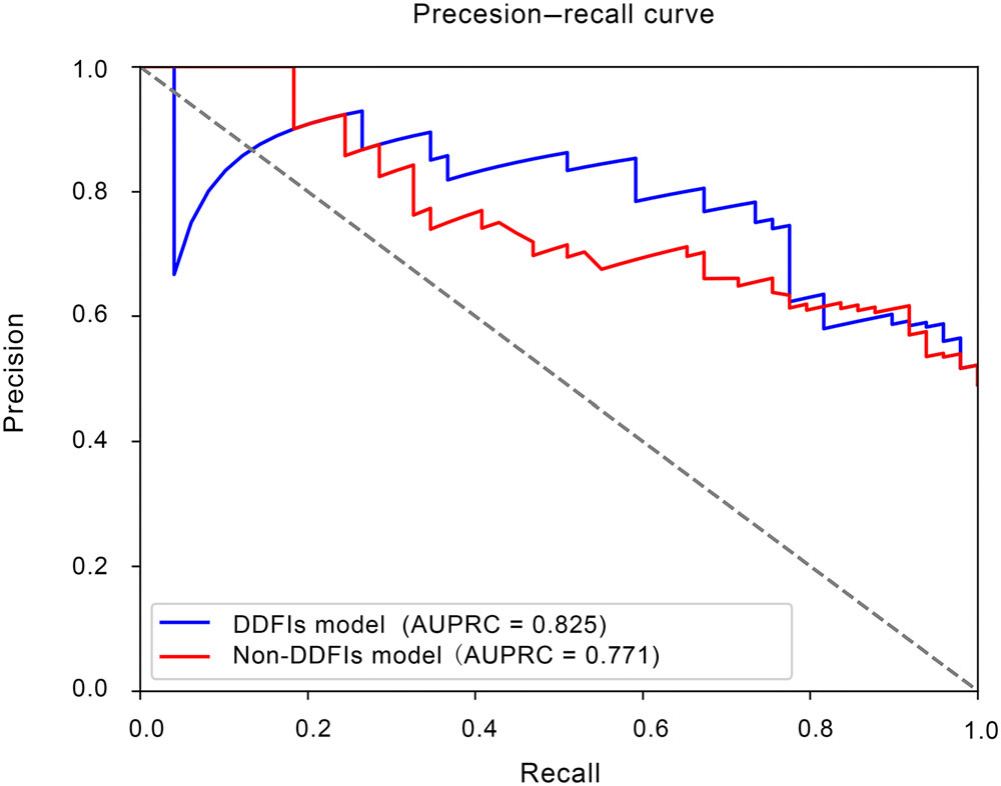
PR curves of the XGBoost model with and without DDFIs. AUPRC, area under the precision–recall curve; PR curve, precession–recall curve.

We first developed a prognostic model incorporating all available variables, which achieved a mean AUROC of 0.708 (95% CI: 0.584–0.832) (Table 2). When DDFIs were excluded, the model yielded a mean AUROC of 0.746 (95% CI: 0.621–0.871). However, the model incorporating DDFIs (specifically mLVs‐SSS wash‐out ratio and AUC) demonstrated the best predictive performance, with a mean AUROC of 0.808 (95% CI: 0.733–0.883) (Table 2, Figure 2B, and Figure S4). This represents an 8.3% improvement in predictive accuracy. Feature importance analysis using XGBoost revealed that postoperative recurrence (0.055), LDH (0.053), MCHC (0.053), chloride (0.053), and MCH (0.053) were the top five predictors in the model including DDFIs (Figure 2C). The mLVs‐SSS wash‐out ratio and AUC had feature importance values of 0.044 and 0.014, respectively. In contrast, the model excluding DDFIs identified plasma chloride (0.061), LDH (0.058), enhancement ratio (0.058), postoperative recurrence (0.055), and γ‐GT (0.054) as the top predictors (Figure 2C). Kaplan–Meier (KM) curves stratified by results of the refined model including DDFIs showed superior discrimination compared to the model excluding these indicators (*p* = 5.66 × 10^−^^8^ vs. *p* = 1.22 × 10^−^^4^) (Figure 2D). Precision–recall (PR) curves further confirmed the enhanced predictive performance of the model with DDFIs, with a mean AUPRC of 0.825 (95% CI: 0.750–0.901) compared to 0.771 (95% CI: 0.646–0.896) for the model without DDFIs (Figure 3). Similar results were observed for progression prediction models. The XGBoost model including DDFIs (AUROC = 0.708 [95% CI: 0.620–0.797]) slightly outperformed the model excluding DDFIs (AUROC = 0.669 [95% CI: 0.554–0.783]) (Table S2, Figure S5, S6). PR curves also supported the improved predictive performance of the model with DDFIs (AUPRC: 0.844 vs. 0.790) (Figure S7). These results underscore the significant contribution of DDFIs in enhancing the predictive accuracy of machine learning models for both prognosis and progression.

**TABLE 2:** The performance of XGBoost models for all.

	All features		Including DDFIs		Excluding DDFIs	
	Mean (SD)	95% CI	Mean (SD)	95% CI	Mean (SD)	95% CI
AUROC	0.708 (0.123)	(0.584, 0.832)	0.808 (0.104)	(0.733, 0.883)	0.746 (0.175)	(0.621, 0.871)
Accuracy	0.680 (0.162)	(0.564, 0.796)	0.760 (0.097)	(0.691, 0.829)	0.690 (0.110)	(0.611, 0.769)
Precision	0.725 (0.196)	(0.585, 0.865)	0.795 (0.127)	(0.704, 0.886)	0.730 (0.168)	(0.610, 0.851)
Recall	0.635 (0.120)	(0.549, 0.721)	0.720 (0.169)	(0.599, 0.841)	0.660 (0.190)	(0.524, 0.796)
F1 score	0.670 (0.136)	(0.573, 0.767)	0.743 (0.100)	(0.671, 0.814)	0.671 (0.119)	(0.586, 0.756)

Abbreviations: AUROC, area under the receiver operating characteristic curve; CI, confidence interval; DDFIs, DCE‐MRI‐based dural drainage function indicators; SD, standard deviation.

### 3.3 DDFIs Enhance Predictive Efficiency of Cox Regression

**FIGURE 4:**
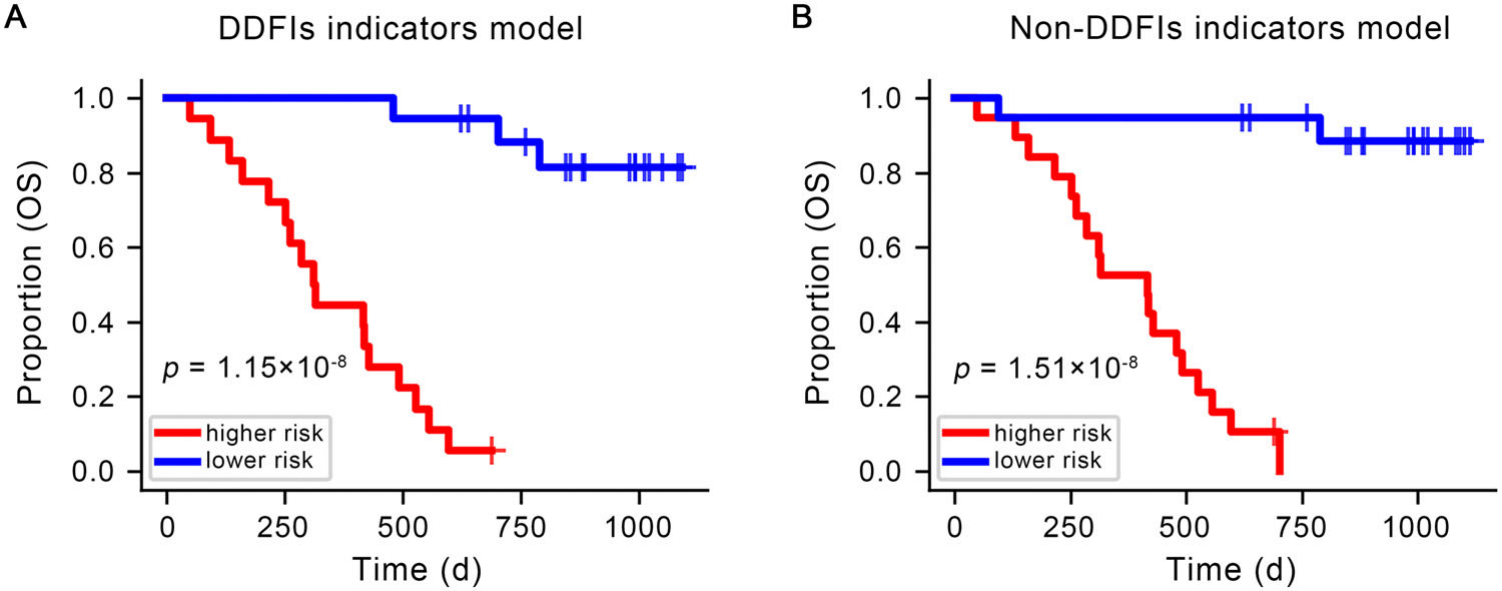
Cox regression model results of all participants. Kaplan–Meier survival curves of the Cox regression model with (left) and without (right) DDFIs. Red curve displayed high risk and blue curve displayed low risk.

Univariate Cox regression analysis revealed a negative correlation between survival time and several variables. The top five variables included LDH, postoperative recurrence, D‐Dimer, γ‐GT, MCHC. Notably, the mLVs‐SSS wash‐out ratio was identified as a protective factor (HR: 0.01, *p* = 0.011). Positive correlations with survival outcomes were observed for chloride (HR: 0.06, *p* = 0.005), platelet‐large cell ratio (P‐LCR) (HR: 0.22, *p* = 0.023), mean platelet volume (MPV) (HR: 0.23, *p* = 0.029), PDW (HR: 0.20, *p *= 0.032), and lymphocyte percentage (HR: 0.21, *p* = 0.038) (Table S3). The multivariate Cox regression model, incorporating significant univariate variables and DDFIs, achieved a concordance index (*c*‐index) of 0.948 (95% CI: 0.946–0.955), compared to 0.919 (95% CI: 0.916–0.940) for the model excluding DDFIs. In the multivariate analysis, the mLVs‐SSS wash‐out ratio lost significance (HR: 0.04, *p* = 0.127). However, shorter survival times were associated with mLVs‐SS wash‐out ratio (HR: 3.01 × 10^4^, *p* = 0.028) and wash‐in rate (HR: 2.82 × 10^8^, *p* = 0.016), while AUC (HR: 1.66 × 10^−^^1^ ^3^, *p* = 0.013) and MPV (HR: 7.16 × 10^−^^14^, *p* = 0.049) were associated with higher survival (Table S4). Other significant risk factors included postoperative recurrence, edema size, LDH, RDW‐SD, PDW, and direct bilirubin. KM curves stratified by the Cox multivariate regression model demonstrated superior discrimination for the model including DDFIs (*p* = 1.15 × 10^−^^8^ vs. *p* = 1.51 × 10^−^^8^) (Figure 4). These findings further validate the role of DDFIs in improving the predictive efficiency of Cox regression models for survival analysis.

### 3.4 DDFIs Enhance Predictive Power of XGBoost in Glioma Subgroup

**FIGURE 5:**
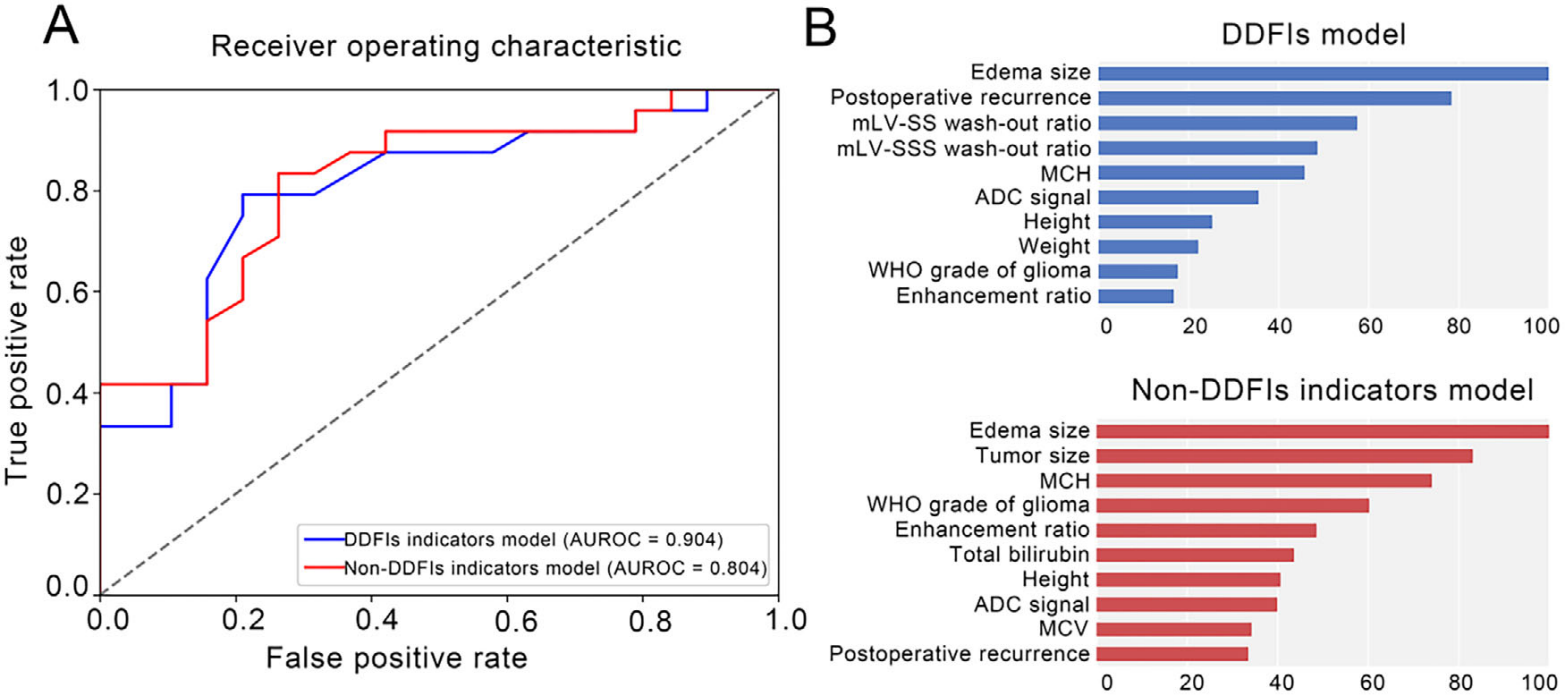
XGBoost Model results of the participants in glioma subgroup. (A) ROC curve of XGBoost model with (blue) and without (red) DDFIs. (B) Top ten important characteristics in the XGBoost model with (left) and without (right) DDFIs. ADC, apparent diffusion coefficient; AUPRC, area under the precision–recall curve; AUROC, area under the receiver operator characteristic curve; MCH, mean corpuscular hemoglobin; MCV, mean corpuscular volume; mLVs, meningeal lymphatic vessels; mLVs‐SS, sigmoid sinus mLVs; mLVs‐SSS, superior sagittal sinus mLVs.

In the glioma subgroup, the model incorporating DDFIs achieved a mean AUROC of 0.904 (95% CI: 0.717–1.000), outperforming the model without DDFIs (mean AUROC = 0.804 [95% CI: 0.629–0.980]) (Figure 5A, Table S5, and Figure S8). The top five features in the model with DDFIs were edema size (0.180), postoperative recurrence (0.141), mLVs‐SS wash‐out ratio (0.104), mLVs‐SSS wash‐out ratio (0.088), and MCH (0.083) (Figure 5B). PR curves further validated the improved predictive performance of the model with DDFIs (AUPRC: 0.950 vs. 0.864) (Figure S9). This subgroup analysis highlights the added value of DDFIs in predicting outcomes specifically for glioma patients, reinforcing their potential as a critical prognostic tool.

## 4 Discussion

In this prospective cohort study, we leveraged machine learning techniques to develop a predictive model for long‐term prognosis in patients with intracranial malignant tumors, building on data from a 2.5‐year follow‐up of our previous cross‐sectional study [21]. The model demonstrated good predictive performance, particularly when incorporating DDFIs. For the entire cohort, the inclusion of DDFIs increased the AUROC from 0.746 to 0.808, while for the glioma subgroup, the AUROC improved from 0.804 to 0.904. These findings underscore the critical role of mLV function in the clinical progression of intracranial malignancies. Furthermore, they highlight the potential of DDFIs as a novel, non‐invasive biomarker for prognostic evaluation and the development of targeted therapeutic strategies, especially in the management of gliomas.

Recent research has emphasized the pivotal role of mLVs in immune surveillance within the central nervous system (CNS), especially in the context of intracranial malignancies [20]. Preclinical studies have shown that mLVs, along with deep cervical lymph nodes (dcLNs), facilitate the drainage of soluble tumor antigens from CNS to dcLNs, where they activate specific T‐cell populations, thereby mediating tumor immunity [17, 26, 27]. Building on this evidence, we hypothesized that mLVs function could serve as an indicator of tumor immunity and correlate with prognosis in patients with intracranial malignant tumors. In our study, mLVs function was categorized into two components: wash‐in function (measured by AUC, TTP, and wash‐in rate) and wash‐out function (measured by mLVs‐SSS and mLVs‐SS wash‐out ratios). Notably, we found that mLVs wash‐out function significantly contributed to the prognostic model, consistent with our earlier findings linking it to tumor radiographic progression [21]. While wash‐in function reflects the short‐term permeability of mLVs and surrounding structures [21, 23], which are often influenced by radiotherapy and chemotherapy [28, 29, 30], wash‐out function represents the efficiency of mLVs in clearing contrast agents over time. This may reflect local vascular damage and impaired drainage capacity due to tumor invasiveness and blood–brain barrier (BBB) disruption [21, 31]. We propose that mLVs wash‐out function, in particular, serves as a prognostic marker by indicating the extent of vascular damage and the mLVs’ ability to drain tumor antigens.

The field of prognosis prediction in malignant tumors has seen significant advancements, with machine learning models achieving impressive performance metrics. For instance, models predicting brain metastasis in breast cancer and non‐small cell lung cancer have reported AUROCs exceeding 0.80 and 0.85, respectively [8, 32].

These models typically rely on a combination of demographics, treatment history, histopathology, genetics (e.g., IDH1 status, 1p/19q co‐deletion, whole‐exome sequencing), transcriptomics (e.g., RNA methylation regulators), and radiomics (e.g., shape, texture, and volumetric features) [8, 33, 34, 35, 36, 37]. Despite their accuracy, the clinical applicability of these models is often hindered by the substantial costs, specialized infrastructure, and long processing times required for multiomics and advanced radiomic analysis. In contrast, our study is the first to propose augmenting basic clinical information with readily available DDFIs as prognostic factors for intracranial tumors, offering a more practical and cost‐effective approach.

To evaluate the reliability of our model, we employed precision–recall (PR) curve analysis, which is particularly informative in datasets with class imbalance. The inclusion of DDFIs improved model performance, resulting in a mean AUPRC of 0.825, compared to 0.771 for the model without DDFIs. While both PR and ROC curves are recommended for evaluating model performance [38], the PR curve is especially useful in imbalanced datasets [39]. In addition, Kaplan–Meier (KM) survival analysis demonstrated better separation between predicted high‐risk and low‐risk groups upon the inclusion of DDFIs, further validating its enhanced prognostic utility. The key findings from the XGBoost model were further cross‐validated using multivariate Cox regression. The addition of DDFIs in the Cox multivariate regression model improved the *c*‐index and enabled more precise risk stratification, as evidenced by the KM curves. Collectively, these results confirmed that DDFIs are critical and reliable predictors of long‐term outcomes in patients with intracranial malignant tumors.

In the XGBoost model integrated with DDFIs, the top five features identified by importance were postoperative recurrence, lactate dehydrogenase (LDH), mean corpuscular hemoglobin concentration (MCHC), chloride, and mean corpuscular hemoglobin (MCH). Postoperative recurrence is a well‐established predictor of poor prognosis in gliomas [40, 41, 42] and other intracranial malignancies [43, 44]. Elevated LDH levels, associated with glioma cell proliferation and invasion [45, 46], have also been linked to poor prognosis in gliomas and brain metastases [47, 48]. Furthermore, our model highlighted the significance of chloride ion dynamics. Mediated by transporters such as NKCC1 and channels like CLIC1 and CLIC4, chloride levels play a critical role in glioma cell volume regulation and tissue invasiveness [49, 50, 51, 52]. Regarding hematological markers, MCH and MCHC levels served as indicators of cancer‐related anemia, which have been previously linked to poor oncological outcome [53, 54, 55], and our findings suggest that these hematological parameters may serve as early indicators of cancer‐related anemia in intracranial tumors. Cox regression analysis further identified postoperative recurrence, mLVs‐SS wash‐out ratio, AUC, wash‐in rate, edema size, LDH, direct bilirubin, RDW‐SD, PDW, and MPV as significant predictors of prognosis. The alignment between the XGBoost feature importance and Cox regression coefficients reinforces the reliability of DDFIs in predicting patient outcomes.

In a subgroup analysis of glioma patients, the model incorporating DDFIs achieved an exceptional AUROC of 0.904, outperforming most previous studies [56, 57, 58, 59]. The top five features in this subgroup were edema size, postoperative recurrence, mLVs‐SS wash‐out ratio, mLVs‐SSS wash‐out ratio, and MCH. Peri‐tumoral edema, a hallmark of glioma [60], has been consistently associated with poor prognosis [61, 62, 63, 64], likely due to its role in increasing intracranial pressure and the risk of brain herniation [65]. We propose that mLVs, as a critical component of the brain's glymphatic system [66], play a key role in fluid exchange between cerebrospinal fluid and interstitial fluid, potentially mitigating edema and its associated risks.

Despite these promising findings, our study has several limitations. First, while increasing evidence supports the visualization of meningeal lymphatic‐related signals via MRI, the precise anatomical origins of these signals and their differentiation from adjacent dural or vascular structures remain subjects of ongoing debate [67]. Therefore, the DDFIs used in this study should be interpreted as functional surrogates of meningeal lymphatic drainage rather than direct anatomical delineations of individual mLVs. Second, the relatively small cohort size necessitates validation in larger, multi‐center, and more diverse populations. Third, the 2.5‐year follow‐up period, while informative, may limit the generalizability of our findings to long‐term survival outcomes. Fourth, the heterogeneity resulting from the inclusion of multiple tumor subtypes may restrict the broader applicability of our results. Finally, while our study highlights the prognostic value of mLVs function, the exact underlying mechanisms remain unclear. Future research should explore how mLVs function influences tumor immunity and prognosis, potentially identifying mLVs as a therapeutic target for intracranial malignancies.

In conclusion, the integration of DDFIs into machine learning models represents a promising approach for enhancing prognostic accuracy in patients with intracranial malignant tumors. This study highlights the potential of mLVs function as a critical, non‐invasive biomarker for prognosis assessment and personalized treatment planning, paving the way for more effective and individualized management of these challenging conditions.

## Author Contributions

M.H.W. and Y.Y.W. conceived and designed the study. L.S.R., W.X.L., L.Y.Y., M.H.W., Y.Y.W., and W.W. contributed to methodology. L.S.R., W.X.L., L.Y.Y., Y.Y.W., W.J.W., G.Y.H., and X.L.Y. collected and managed data. L.S.R., W.X.L., and L.Y.Y. performed statistical analyses. W.X.L., Y.Q.H., S.B.X., J.Y.Z., F.L., and X.P.S. validated results. L.S.R., W.X.L., and L.Y.Y. drafted the manuscript. M.H.W. acquired funding. M.H.W. and Y.Y.W. supervised the research. All authors critically revised the manuscript and approved the final version.

## Funding

The clinical study (Registration ID: ChiCTR2400088215) was funded through the National Key Research and Development Program of China (Program ID: 2021YFC2502200) Hubei Provincial Natural Science Foundation (Program ID: 2022CFA097).

## Ethics Statement

This prospective cohort study was registered with Chinese Clinical Trial Registry (Registration number: ChiCTR2400088215), and approved by the Medical Ethics Committee of Tongji Hospital, Tongji Medical College, Huazhong University of Science and Technology (TJ‐IRB20220456).

## Conflicts of Interest

The authors declare no conflicts of interest.

## Supporting information




**Supporting file**: mco270992‐sup‐0001‐SuppMat.docx.

## Data Availability

The data supporting the findings of this study are not publicly available but are securely stored at Tongji Hospital. Access to the data may be granted upon reasonable request, subject to approval by the research team and the ethics committee. Requests for the original data should be directed to the corresponding author. Additional supporting information can be found in the supporting files.
