# Supplementary material for: Integrating DCE‐MRI‐Based Dural Drainage Function Indicators into Machine Learning Models for Improved Intracranial Tumor Prognosis
Source: MedComm (2020). 2026 Sep 25;7(10):e70992. doi: 10.1002/mco2.70992 (PMC13613099; doi:10.1002/mco2.70992)
Supplement: Supplementary file 1 [file MCO2-7-e70992-s001.docx]

**Supplemental Materials**

Title page.	2

Table S1. The performance of XGBoost models on intracranial tumor progression.	3

Table S2. Multivariate Cox regression analysis including mLVs indicators.	4

Table S3. Information-criterion comparison of Cox models excluding and including mLVs indicators in the matched complete-case dataset.	5

Table S4. The performance of XGBoost models for the participants in glioma subgroup.	6

Table S5. The performance of GBDT models for the participants.	7

Table S6. Univariate Cox regression analysis.	8

Figure S1. Ten-fold cross-validation results of the XGBoost model for all participants.	11

Figure S2. XGBoost model results on intracranial tumor progression.	12

Figure S3. Ten-fold cross-validation results of the XGBoost model on intracranial tumor progression for all participants.	13

Figure S4. PR curves of the XGBoost model on intracranial tumor progression with and without mLVs indicators.	14

Figure S5. Ten-fold cross-validation results of the XGBoost model for the participants in glioma subgroup.	15

Figure S6. PR curves of the XGBoost model with and without mLVs indicators in glioma subgroup.	16

**Integrating Dynamic Contrast-enhanced Magnetic Resonance Imaging Based Dural Drainage Function Indicators into Machine Learning Models for Improved Intracranial Tumor Prognosis**

Lusen Ran^1,10#^, Wenxi Luo^2#^, Luyun You^3,1#^, Wenjie Wei^4^, Yuqin He^1^, Shabei Xu^1^, Jiayu Zhu^5,6,7^, Fan Long^5^, Xiaopeng Song^5,8^, Guangyuan Hu^4^, Xianglin Yuan^4^, Wei Wang^1^, Feng Lu^9^, Minghuan Wang^1*^, YingYing Wu^4*^

^1^ Department of Neurology, Tongji Hospital, Tongji Medical College, Huazhong University of Science and Technology, Wuhan, China | ^2^ College of Life Science and Technology, Huazhong University of Science and Technology, Wuhan, China | ^3^ Department of Neurology, The Second Affiliated Hospital of Nanchang University, Nanchang, Jiangxi, China | ^4^ Department of Oncology, Tongji Hospital, Tongji Medical College, Huazhong University of Science and Technology, Wuhan, China | ^5^ Central Research Institute, United Imaging Healthcare Group, Shanghai, China | ^6^ Paul C. Lauterbur Research Centre for Biomedical Imaging, Shenzhen Institute of Advanced Technology, Chinese Academy of Science, Shenzhen, Guangdong, China | ^7^ Shenzhen College of Advanced Technology, University of Chinese Academy of Sciences, Shenzhen, Guangdong, China | ^8^ Wuhan Zhongke Industrial Research Institute of Medical Science, Wuhan, China | ^9^ National Engineering Research Center for Big Data Technology and System, Services Computing Technology and System Lab, Cluster and Grid Computing Lab, School of Computer Science and Technology, Huazhong University of Science and Technology, Wuhan, China | ^10^ Shenzhen Nanshan People’s Hospital, Shenzhen, China

^#^These authors contributed equally to this work.

^*^**Correspondence**: YingYing Wu (2016TJ0250@hust.edu.cn) | Minghuan Wang ( mhwang@tjh.tjmu.edu.cn)

**Funding:** The clinical study (Registration ID: ChiCTR2400088215) was supported by the Hubei Provincial International Science and Technology Cooperation Project under the Hubei Provincial Science and Technology Plan (Grant No. 2025EHA036).

| **Table S1. The performance of XGBoost models on intracranial tumor progression.** | | | | | | |
| --- | --- | --- | --- | --- | --- | --- |
|  | **All features** | | **Including mLVs indicators** | | **Excluding mLVs indicators** | |
|  | **Mean(SD)** | **95% CI** | **Mean(SD)** | **95% CI** | **Mean(SD)** | **95% CI** |
| **AUROC** | 0.708(0.184) | (0.576, 0.840) | 0.708(0.123) | (0.620, 0.797) | 0.669(0.160) | (0.554,0.783) |
| **Accuracy** | 0.697(0.116) | (0.614, 0.780) | 0.687(0.120) | (0.601, 0.772) | 0.697(0.095) | (0.629, 0.765) |
| **Precision** | 0.716(0.098) | (0.645, 0.786) | 0.699(0.097) | (0.630, 0.768) | 0.690(0.082) | (0.631, 0.748) |
| **Recall** | 0.888(0.188) | (0.754, 1.022) | 0.917(0.162) | (0.801, 1.033) | 0.967(0.070) | (0.916, 1.017) |
| **F1 score** | 0.780(0.106) | (0.705, 0.855) | 0.782(0.099) | (0.712, 0.853) | 0.802(0.063) | (0.757, 0.847) |
| Abbreviations: AUROC, Area Under the Receiver Operating Characteristic curve; mLVs, Meningeal Lymphatic Vessels; SD, Standard deviation. | | | | | | |

| **Table S2.** **Multivariate Cox regression analysis including mLVs indicators.** | | | | | |
| --- | --- | --- | --- | --- | --- |
| **Features** | **Coefficient** | **HR** | **95% CI of HR** | | ***p*** |
|  |  |  | **Lower** | **Upper** |  |
| RDW-SD, fL | 12.62 | 3.03×10^5^ | 1.83×10^2^ | 5.04×10^8^ | 0.001 |
| PDW, fL | 25.53 | 1.22×10^11^ | 3.61×10^3^ | 4.13×10^18^ | 0.004 |
| Postoperative recurrence | 4.08 | 59.14 | 3.64 | 961.64 | 0.004 |
| AUC | -29.43 | 1.66×10^-13^ | 1.53×10^-23^ | 1.80×10^-3^ | 0.013 |
| Wash-in rate, A.U. / s | 19.46 | 2.82×10^8^ | 4.03×10^1^ | 1.97×10^15^ | 0.016 |
| Edema size, mm^3^ | 15.49 | 5.31×10^6^ | 1.79×10^1^ | 1.58×10^12^ | 0.016 |
| mLV-SS wash-out ratio | 10.31 | 3.01×10^4^ | 3.04 | 2.97×10^8^ | 0.028 |
| Direct bilirubin, μmol/L | 48.46 | 1.12×10^21^ | 1.32×10^2^ | 9.43×10^39^ | 0.029 |
| LDH,U/L | 51.88 | 3.39×10^22^ | 7.19 | 1.60×10^44^ | 0.042 |
| MPV, fL | -30.27 | 7.16×10^-14^ | 5.60×10^-27^ | 9.16×10^-1^ | 0.049 |
| TTP | 6.93 | 1.02×10^3^ | 9.50×10^-1^ | 1.10×10^6^ | 0.052 |
| Total bilirubin,μmol/L | -16.33 | 8.09×10^-8^ | 1.62×10^-16^ | 4.05×10^1^ | 0.110 |
| mLV-SSS wash-out ratio | -3.21 | 4.04×10^-2^ | 6.55×10^-4^ | 2.49 | 0.127 |
| D-Dimer, μg/ml FEU | -26.10 | 4.61×10^-12^ | 1.24×10^-26^ | 1.72×10^3^ | 0.127 |
| Sex | 2.25 | 9.49 | 0.48 | 188.04 | 0.140 |
| WHO grade of glioma | 1.01 | 2.74 | 0.71 | 10.66 | 0.145 |
| Lymphocyte percentage,% | 5.06 | 1.57×10^2^ | 8.02×10^-2^ | 3.07×10^5^ | 0.191 |
| MCHC, g/L | 10.36 | 3.16×10^4^ | 9.52×10^-5^ | 1.05×10^13^ | 0.301 |
| Chloride, mmol/L | 9.25 | 1.04×10^4^ | 3.74×10^-5^ | 2.88×10^12^ | 0.351 |
| MCV, fL | 16.68 | 1.76×10^7^ | 1.75×10^-13^ | 1.77×10^27^ | 0.478 |
| MCH, pg | -14.53 | 4.89×10^-7^ | 5.24×10^-31^ | 4.56×10^17^ | 0.606 |
| γ-GT,U/L | 2.29 | 9.91 | 8.61×10^-5^ | 1.14×10^6^ | 0.700 |
| P-LCR,% | -1.26 | 2.84×10^-1^ | 8.91×10^-15^ | 9.07×10^12^ | 0.937 |
| Abbreviations: AUC, Area Under the Curve; HR, Hazard Ratio; LDH, Lactate Dehydrogenase; MCH, Mean Corpuscular Hemoglobin; MCHC, Mean Corpuscular Hemoglobin Concentration; MCV, Mean Corpuscular Volume; mLVs-SS, Sigmoid Sinus mLVs; mLVs-SSS, Superior Sagittal Sinus mLVs; P-LCR, Platelet Large Cell Ratio; TTP, Time to Peak; γ-GT, Gamma-Glutamyl Transferase. | | | | | |

| **Table S3 Information-criterion comparison of Cox models excluding and including mLVs indicators in the matched complete-case dataset.** | | | | |
| --- | --- | --- | --- | --- |
| **Model** | **c-index** | **Log partial likelihood** | **Partial AIC** | **BIC** |
| **Excluding mLVs indicators** | 0.907 | -59.423 | 154.846 | 183.349 |
| **Including mLVs indicators** | 0.903 | -58.982 | 163.965 | 200.385 |
| Abbreviations: AIC, Akaike information criterion; BIC, Bayesian information criterion mLVs, Meningeal Lymphatic Vessels. | | | | |

| **Table S4. The performance of XGBoost models for the participants in Glioma Subgroup.** | | | | | | |
| --- | --- | --- | --- | --- | --- | --- |
|  | **All variables** | | **Including mLVs-related variables** | | **Excluding mLVs-related variables** | |
|  | **Mean (SD)** | **95% CI** | **Mean (SD)** | **95% CI** | **Mean (SD)** | **95% CI** |
| **AUROC** | 0.775(0.343) | (0.530,1.020) | 0.904(0.262) | (0.717, 1.092) | 0.804(0.245) | (0.629,0.980) |
| **Accuracy** | 0.675(0.287) | (0.470, 0.880) | 0.770(0.226) | (0.608, 0.932) | 0.765(0.168) | (0.645, 0.885) |
| **Precision** | 0.760(0.319) | (0.532, 0.988) | 0.877(0.202) | (0.732, 1.022) | 0.792(0.189) | (0.656, 0.927) |
| **Recall** | 0.717(0.352) | (0.465, 0.968) | 0.767(0.263) | (0.579, 0.955) | 0.850(0.200) | (0.707, 0.993) |
| **F1 score** | 0.698(0.288) | (0.493, 0.904) | 0.788(0.188) | (0.654, 0.923) | 0.802(0.146) | (0.698, 0.907) |
| Abbreviations: AUROC, Area Under the Receiver Operating Characteristic curve; mLVs, Meningeal Lymphatic Vessels; SD, Standard deviation. | | | | | | |

| **Table S5 The performance of GBDT models for the participants.** | | | | | | | |
| --- | --- | --- | --- | --- | --- | --- | --- |
| **Model** | **Feature Type** | **AUROC** | **Precision** | **Recall** | **F1 score** | **Accuracy** | **AUPRC** |
| **All participants** | | | | | | | |
| XGBoost | mLVs | 0.808 | 0.703 | 0.715 | 0.703 | 0.710 | 0.825 |
|  | non-mLVs | 0.756 | 0.647 | 0.715 | 0.668 | 0.650 | 0.771 |
| LightGBM | mLVs | 0.723 | 0.663 | 0.655 | 0.654 | 0.670 | 0.719 |
|  | non-mLVs | 0.687 | 0.650 | 0.655 | 0.647 | 0.660 | 0.646 |
| CatBoost | mLVs | 0.651 | 0.605 | 0.510 | 0.527 | 0.580 | 0.678 |
|  | non-mLVs | 0.679 | 0.682 | 0.555 | 0.575 | 0.640 | 0.728 |
| **Intracranial tumor progression** | | | | | | | |
| XGBoost | mLVs | 0.708 | 0.705 | 0.967 | 0.812 | 0.717 | 0.844 |
|  | non-mLVs | 0.669 | 0.670 | 0.907 | 0.764 | 0.657 | 0.790 |
| LightGBM | mLVs | 0.650 | 0.700 | 0.779 | 0.733 | 0.647 | 0.768 |
|  | non-mLVs | 0.661 | 0.705 | 0.767 | 0.730 | 0.647 | 0.763 |
| CatBoost | mLVs | 0.610 | 0.702 | 0.893 | 0.782 | 0.687 | 0.762 |
|  | non-mLVs | 0.616 | 0.688 | 0.876 | 0.766 | 0.667 | 0.790 |
| **Glioma subgroup** | | | | | | | |
| XGBoost | mLVs | 0.904 | 0.843 | 0.833 | 0.805 | 0.775 | 0.950 |
|  | non-mLVs | 0.804 | 0.710 | 0.800 | 0.720 | 0.695 | 0.864 |
| LightGBM | mLVs | 0.500 | 0.555 | 1.000 | 0.711 | 0.555 | 0.778 |
|  | non-mLVs | 0.500 | 0.555 | 1.000 | 0.711 | 0.555 | 0.778 |
| CatBoost | mLVs | 0.808 | 0.758 | 0.783 | 0.749 | 0.715 | 0.853 |
|  | non-mLVs | 0.733 | 0.708 | 0.733 | 0.706 | 0.685 | 0.821 |
| Abbreviations: AUROC, Area Under the Receiver Operating Characteristic curve; AUPRC, area under the precision-recall curve; Meningeal Lymphatic Vessels. | | | | | | | |

| **Table S6. Univariate Cox regression analysis.** | | | | | | |  |
| --- | --- | --- | --- | --- | --- | --- | --- |
| **Features** | **Coefficient** | **HR** | **95% CI of HR** | | ***p*** | **Bonferroni *p*** | **FDR *p*** |
|  |  |  | **Lower** | **Upper** |  |  |  |
| LDH,U/L | 4.21 | 67.21 | 8.24 | 548.17 | <0.001 | 0.007 | 0.007 |
| Postoperative recidivation | 1.45 | 4.26 | 1.95 | 9.33 | <0.001 | 0.023 | 0.012 |
| D-Dimer, μg/ml FEU | 4.36 | 78.42 | 5.80 | 1059.70 | 0.001 | 0.084 | 0.028 |
| γ-GT,U/L | 3.20 | 24.49 | 2.86 | 209.91 | 0.004 | 0.289 | 0.064 |
| MCHC, g/L | 2.28 | 9.79 | 2.08 | 46.06 | 0.004 | 0.318 | 0.064 |
| Chloride, mmol/L | -2.82 | 0.06 | 0.01 | 0.42 | 0.005 | 0.388 | 0.065 |
| MCH, pg | 3.01 | 20.24 | 2.39 | 171.50 | 0.006 | 0.476 | 0.068 |
| mLV-SSS wash-out ratio | -2.19 | 0.11 | 0.02 | 0.61 | 0.011 | 0.901 | 0.112 |
| WHO grade of glioma | 0.63 | 1.87 | 1.15 | 3.05 | 0.012 | 1.000 | 0.112 |
| Sex | 0.75 | 2.12 | 1.14 | 3.96 | 0.018 | 1.000 | 0.150 |
| P-LCR,% | -1.53 | 0.22 | 0.06 | 0.81 | 0.023 | 1.000 | 0.172 |
| MPV, fL | -1.46 | 0.23 | 0.06 | 0.86 | 0.029 | 1.000 | 0.183 |
| PDW, fL | -1.60 | 0.20 | 0.05 | 0.87 | 0.032 | 1.000 | 0.183 |
| Direct bilirubin, μmol/L | 1.70 | 5.49 | 1.13 | 26.74 | 0.035 | 1.000 | 0.183 |
| Edema size, mm3 | 1.61 | 5.00 | 1.11 | 22.50 | 0.036 | 1.000 | 0.183 |
| RDW-SD, fL | 1.30 | 3.69 | 1.08 | 12.53 | 0.037 | 1.000 | 0.183 |
| Lymphocyte percentage,% | -1.56 | 0.21 | 0.05 | 0.92 | 0.038 | 1.000 | 0.183 |
| MCV, fL | 2.14 | 8.54 | 1.05 | 69.32 | 0.045 | 1.000 | 0.195 |
| Total bilirubin, μmol/L | 1.33 | 3.79 | 1.03 | 13.95 | 0.045 | 1.000 | 0.195 |
| Sodium, mmol/L | -2.28 | 0.10 | 0.01 | 1.08 | 0.058 | 1.000 | 0.230 |
| Basophil percentage,% | -1.73 | 0.18 | 0.03 | 1.07 | 0.059 | 1.000 | 0.230 |
| mLV-SS wash-out ratio | -1.16 | 0.31 | 0.08 | 1.23 | 0.096 | 1.000 | 0.346 |
| Neutrophil percentage,% | 1.20 | 3.30 | 0.80 | 13.66 | 0.099 | 1.000 | 0.346 |
| Monocyte count,10^9^/L | 1.10 | 2.99 | 0.81 | 11.12 | 0.101 | 1.000 | 0.346 |
| Creatinine, μmol/L | 1.25 | 3.49 | 0.71 | 17.16 | 0.124 | 1.000 | 0.407 |
| Neutrophil count,10^9^/L | 0.96 | 2.60 | 0.75 | 9.08 | 0.133 | 1.000 | 0.413 |
| Radiation therapy | -0.46 | 0.63 | 0.35 | 1.16 | 0.136 | 1.000 | 0.413 |
| Height, m | 0.99 | 2.69 | 0.67 | 10.80 | 0.162 | 1.000 | 0.474 |
| Indirect bilirubin, μmol/L | 0.81 | 2.25 | 0.71 | 7.17 | 0.171 | 1.000 | 0.483 |
| PVS in the midbrain | 0.43 | 1.53 | 0.78 | 3.00 | 0.212 | 1.000 | 0.579 |
| Leukocyte count,10^9^/L | 0.78 | 2.18 | 0.60 | 7.96 | 0.239 | 1.000 | 0.631 |
| Basophil count,10^9^/L | -1.15 | 0.32 | 0.04 | 2.36 | 0.261 | 1.000 | 0.669 |
| Lymphocyte count,10^9^/L | -0.73 | 0.48 | 0.12 | 1.87 | 0.290 | 1.000 | 0.709 |
| RDW-CV | 0.66 | 1.94 | 0.56 | 6.65 | 0.294 | 1.000 | 0.709 |
| Bicarbonate, mmol/L | -0.69 | 0.50 | 0.13 | 1.90 | 0.310 | 1.000 | 0.727 |
| Erythrocyte count,10^12^/L | -0.78 | 0.46 | 0.09 | 2.40 | 0.356 | 1.000 | 0.797 |
| AST,U/L | 0.91 | 2.48 | 0.34 | 18.33 | 0.373 | 1.000 | 0.797 |
| Chemotherapy-induced leukopenia | 0.34 | 1.40 | 0.66 | 2.98 | 0.381 | 1.000 | 0.797 |
| Total cholesterol, mmol/L | -0.51 | 0.60 | 0.19 | 1.90 | 0.388 | 1.000 | 0.797 |
| ALT,U/L | 0.84 | 2.33 | 0.30 | 18.09 | 0.420 | 1.000 | 0.797 |
| TTP, s | 0.46 | 1.59 | 0.50 | 5.08 | 0.435 | 1.000 | 0.797 |
| Monocyte percentage,% | 0.51 | 1.67 | 0.45 | 6.16 | 0.439 | 1.000 | 0.797 |
| AUC, A.U. × s | 0.53 | 1.70 | 0.44 | 6.61 | 0.443 | 1.000 | 0.797 |
| Multiple or Single tumor | 0.24 | 1.28 | 0.68 | 2.41 | 0.451 | 1.000 | 0.797 |
| INR | 0.57 | 1.77 | 0.39 | 8.10 | 0.463 | 1.000 | 0.797 |
| Fibrinogen, g/L | 0.56 | 1.75 | 0.38 | 8.11 | 0.477 | 1.000 | 0.797 |
| BMI, kg/m^2^ | -0.55 | 0.58 | 0.12 | 2.80 | 0.497 | 1.000 | 0.797 |
| PT,s | 0.56 | 1.74 | 0.35 | 8.73 | 0.500 | 1.000 | 0.797 |
| PVS in the semi-ovoid body | 0.12 | 1.12 | 0.80 | 1.58 | 0.511 | 1.000 | 0.797 |
| Plateletcrit, % | -0.49 | 0.61 | 0.14 | 2.68 | 0.516 | 1.000 | 0.797 |
| Potassium, mmol/L | 0.55 | 1.73 | 0.33 | 9.00 | 0.516 | 1.000 | 0.797 |
| Calcium, mmol/L | 0.51 | 1.66 | 0.36 | 7.73 | 0.518 | 1.000 | 0.797 |
| SBP, mmHg | -0.41 | 0.66 | 0.18 | 2.46 | 0.537 | 1.000 | 0.797 |
| Total protein, g/L | 0.39 | 1.48 | 0.41 | 5.37 | 0.548 | 1.000 | 0.797 |
| ADC signal | 0.55 | 1.73 | 0.28 | 10.69 | 0.554 | 1.000 | 0.797 |
| Albumin, g/L | 0.46 | 1.58 | 0.32 | 7.83 | 0.575 | 1.000 | 0.797 |
| DBP, mmHg | -0.51 | 0.60 | 0.10 | 3.57 | 0.575 | 1.000 | 0.797 |
| Wash-in rate, A.U. / s | 0.39 | 1.47 | 0.38 | 5.70 | 0.575 | 1.000 | 0.797 |
| Enhancement ratio | -0.40 | 0.67 | 0.16 | 2.87 | 0.589 | 1.000 | 0.797 |
| PA,% | -0.41 | 0.67 | 0.15 | 3.06 | 0.601 | 1.000 | 0.797 |
| TT,s | 0.45 | 1.57 | 0.29 | 8.55 | 0.602 | 1.000 | 0.797 |
| PVS in the hippocampus | 0.15 | 1.16 | 0.66 | 2.06 | 0.603 | 1.000 | 0.797 |
| Hemoglobin, g/L | 0.39 | 1.48 | 0.28 | 7.84 | 0.648 | 1.000 | 0.838 |
| Brain tumor | -0.16 | 0.85 | 0.42 | 1.75 | 0.666 | 1.000 | 0.838 |
| Eosinophil percentage,% | 0.43 | 1.54 | 0.20 | 11.57 | 0.677 | 1.000 | 0.838 |
| Urea, mmol/L | 0.32 | 1.37 | 0.28 | 6.64 | 0.695 | 1.000 | 0.838 |
| Age, year | 0.23 | 1.26 | 0.38 | 4.22 | 0.704 | 1.000 | 0.838 |
| Corrected calcium, mmol/L | 0.29 | 1.34 | 0.30 | 5.94 | 0.704 | 1.000 | 0.838 |
| PVS in the basal ganglia | -0.12 | 0.89 | 0.48 | 1.63 | 0.705 | 1.000 | 0.838 |
| Tumor size, mm^3^ | 0.30 | 1.35 | 0.24 | 7.50 | 0.729 | 1.000 | 0.851 |
| Globulin, g/L | 0.27 | 1.32 | 0.27 | 6.50 | 0.737 | 1.000 | 0.851 |
| ALP,U/L | 0.27 | 1.31 | 0.25 | 6.74 | 0.748 | 1.000 | 0.852 |
| Eosinophil count, 10^9^/L | 0.29 | 1.34 | 0.18 | 9.78 | 0.772 | 1.000 | 0.862 |
| Uric acid, μmol/L | -0.23 | 0.80 | 0.15 | 4.19 | 0.787 | 1.000 | 0.862 |
| Weight, kg | 0.18 | 1.19 | 0.33 | 4.35 | 0.789 | 1.000 | 0.862 |
| eGFR | -0.20 | 0.82 | 0.16 | 4.20 | 0.807 | 1.000 | 0.871 |
| Platelet count,10^9^/L | 0.15 | 1.16 | 0.32 | 4.17 | 0.819 | 1.000 | 0.872 |
| Hematocrit,% | -0.11 | 0.89 | 0.17 | 4.76 | 0.894 | 1.000 | 0.937 |
| APTT,s | 7.20×10^-2^ | 1.07 | 0.32 | 3.58 | 0.907 | 1.000 | 0.937 |
| Prior surgery | 3.52×10^-2^ | 1.04 | 0.52 | 2.06 | 0.920 | 1.000 | 0.937 |
| Chemotherapy | 3.01×10^-2^ | 1.03 | 0.55 | 1.93 | 0.925 | 1.000 | 0.937 |
| Systemic therapies | 2.47×10^-4^ | 1.00 | 0.49 | 2.05 | 0.999 | 1.000 | 0.999 |
| Abbreviations: ADC, Apparent Diffusion Coefficient; ALP, Alkaline Phosphatase; ALT, Alanine Aminotransferase; APTT, Activated partial thromboplastin time; AUC, Area Under the Curve; BMI, Body Mass Index; DBP, Diastolic Blood Pressure; eGFR, estimated Glomerular Filtration Rate; HR, Hazard Ratio; INR, International normalized ratio; LDH, Lactate Dehydrogenase; MCH, Mean Corpuscular Hemoglobin; MCHC, Mean Corpuscular Hemoglobin Concentration; MCV, Mean Corpuscular Volume; mLVs-SS, Sigmoid Sinus mLVs; mLVs-SSS, Superior Sagittal Sinus mLVs; MPV, Mean Platelet Volume; PA, Prealbumin; PDW, Platelet Distribution Width; P-LCR, Platelet Large Cell Ratio; PT, Prothrombin Time; PVS, Perivascular Space; RDW-CV, Red Cell Distribution Width - Coefficient of Variation; RDW-SD, Red Cell Distribution Width - Standard Deviation; SBP, Systolic Blood Pressure; TTP, Time to Peak; TT, Thrombin Time; γ-GT, Gamma-Glutamyl Transferase; WHO, World Health Organization. | | | | | | | |

**Figure S1.** **Ten-fold cross-validation results of the XGBoost model for all participants.**

Abbreviations: AUROC, Area Under the Receiver Operating Characteristic curve; mLVs, Meningeal Lymphatic Vessels; Std, Standard deviation.

**Figure S2. XGBoost model results on intracranial tumor progression**

(A) ROC curve of XGBoost model with (blue) and without (red) mLVs indicators. (B) Top ten importance of the characteristics in XGBoost model with (left) and without (right) mLVs indicators. Abbreviations: AST, Aspartate Aminotransferase; AUC, Area Under the Curve; AUROC, Area Under the Receiver Operating Characteristic curve; LDH, Lactate Dehydrogenase; mLVs, Meningeal Lymphatic Vessels; mLVs-SSS, Superior Sagittal Sinus mLVs; RDW-SD, Red Cell Distribution Width - Standard Deviation; Std, Standard deviation; TT, Thrombin Time; γ-GT, Gamma-Glutamyl Transferase.

**Figure S3. Ten-fold cross-validation results of the XGBoost model on intracranial tumor progression for all participants.**

Abbreviations: AUROC, Area Under the Receiver Operating Characteristic curve; mLVs, Meningeal Lymphatic Vessels; Std, Standard deviation.

**Figure S4. PR curves of the XGBoost model on intracranial tumor progression with and without mLVs indicators.**

Abbreviations: AUPRC, Area Under the Precision-Recall Curve; mLVs, Meningeal Lymphatic Vessels; PR curve, Precession-Recall curve; Std, Standard deviation.

**Figure S5. Ten-fold cross-validation results of the XGBoost model for the participants in glioma subgroup.**

Abbreviations: AUROC, Area Under the Receiver Operating Characteristic curve; mLVs, Meningeal Lymphatic Vessels; Std, Standard deviation.

**Figure S6. PR Curves of the XGBoost Model with and without mLVs indicators in glioma subgroup.**

Abbreviations: AUPRC, Area Under the Precision-Recall Curve; mLVs, Meningeal Lymphatic Vessels; PR curve, Precession-Recall curve; Std, Standard deviation.
